# Health problems and help-seeking in a nationwide sample of operational Norwegian ambulance personnel

**DOI:** 10.1186/1471-2458-8-3

**Published:** 2008-01-04

**Authors:** Tom Sterud, Erlend Hem, Øivind Ekeberg, Bjørn Lau

**Affiliations:** 1 Department of Behavioural Sciences in Medicine, Institute of Basic Medical Sciences, Faculty of Medicine, University of Oslo, PO Box 1111 Blindern, NO-0317 Oslo, Norway; 2 National Institute of Occupational Health, Oslo, Norway

## Abstract

**Background:**

To estimate the prevalence of anxiety and depression symptoms, and their association with professional help-seeking, among operational ambulance personnel and a general working population, and to study the symptoms of musculoskeletal pain and disturbed sleep among ambulance personnel.

**Methods:**

The results of a comprehensive nationwide questionnaire survey of operational ambulance personnel (n = 1180) were compared with the findings of a population-based Norwegian health study of working people (n = 31,987). The questionnaire included measures of help-seeking, the Hospital Anxiety and Depression Scale, the Subjective Health Complaints Questionnaire, the Karolinska Sleep Questionnaire and the Need for Recovery after Work Scale.

**Results:**

Compared with those in the reference population, the mean of level anxiety symptoms in the ambulance sample was lower for men (3.5 vs. 3.9, *P <*0.001) and women (4.0 vs. 4.4, *P <*0.05), and the mean level of depression symptoms in ambulance workers was lower for men (2.3 vs. 2.8, *P <*0.05) but not for women (2.9 vs. 3.1, *P = *0.22). A model adjusted for anxiety and depression symptoms indicated that ambulance personnel had lower levels of help-seeking except for seeing a chiropractor (12% vs. 5%, *P <*0.01). In the ambulance sample, symptoms of musculoskeletal pain were most consistently associated with help-seeking. In the adjusted model, only symptoms of disturbed sleep were associated with help-seeking from a psychologist/psychiatrist (total sample = 2.3%). Help-seeking was more often reported by women but was largely unaffected by age.

**Conclusion:**

The assumption that ambulance personnel have more anxiety and depression symptoms than the general working population was not supported. The level of musculoskeletal pain and, accordingly, the level of help-seeking from a chiropractor were higher for ambulance workers. More research should address the physical strains among ambulance personnel.

## Background

Some previous studies have reported a high prevalence of mental [1,2] and physical [3] health problems among ambulance workers. However, it is unclear whether ambulance personnel have more mental health problems than the general working population. Furthermore, the relationship between health problems and help-seeking among ambulance personnel has not been studied [4].

The help-seeking literature has reported that whether people seek help is associated with needs, socio-demographic factors and attitudes [5]. Among health care workers there may be a general reluctance towards seeking professional help, especially for mental health problems, as has been reported for Norwegian physicians [6]. Several reasons may account for the supposed resistance. Health care workers, including ambulance personnel, are trained to provide medical care under life-and-death circumstances and to control their emotions and deal with other people's problems while on the job. Therefore, it may be difficult for ambulance personnel to admit to having personal problems and so, rather than seeking help, they may try to solve their problems alone. Further, the male-dominated nature of the ambulance occupation may promote a culture that discourages people from admitting that they are having mental problems, which could lead to under-recognition and a low propensity to seek help [7]. In a study of police, another male-dominated occupation, employees reported and sought professional help for somatic complaints more often than did the general population, but their threshold for contacting a psychologist or a psychiatrist was reported to be high [8]. A similar help-seeking profile may be expected for ambulance personnel.

The type of symptoms experienced influences the decision to seek professional help or not. People are more likely to seek help for somatic complaints, whereas most persons with mental disorders do not get professional help [9]. Moreover, somatic symptoms such as fatigue, musculoskeletal pain and sleep problems are common in both anxiety and depression [10,11], and people with both affective disorders and painful physical symptoms have a significantly lower rate of help-seeking for emotional reasons [12].

The co-morbidity of mental health problems and somatic complaints has not been studied in the ambulance services. In general, few studies have investigated the physical health of ambulance workers, but it has been suggested that they have more self-reported musculoskeletal [13] and physical health [3] problems than the general population. Further, there has been a lack of alternative health measures to identify smaller, everyday problems in ambulance workers, such as disturbed sleep and the need for recovery after work [4]. One obvious source of disturbed sleep is the effect of work life, such as shift work, which is an integral part of ambulance work [14].

Against this background, we conducted a study to address the level of mental and physical health problems and the corresponding level of help-seeking in a nationwide sample of operational ambulance personnel. Reference material on depression, anxiety and help-seeking was obtained from a general working population in Norway. We developed the following hypotheses.

1. Ambulance personnel have more anxiety and depression symptoms than the general working population, and they are less likely to seek help than the general population, and this may be influenced by the level of depression and anxiety symptoms.

2. Ambulance personnel report a relatively high level of somatic complaints. The prevalence of symptoms of musculoskeletal pain, disturbed sleep and need for recovery is higher among operational ambulance personnel with anxiety and depression symptoms.

3. Ambulance personnel (like police) are more likely to seek help for somatic complaints and are less likely to seek help for mental problems. There is a relationship between different types of health problems and the type of professional help that is sought.

## Methods

### Ambulance sample

Ambulance services in Norway are organized into 19 main ambulance regions that are responsible for ensuring adequate ambulance services for all communities. Some hospitals have their own integrated ambulance departments, whereas, in several counties, ambulance services are organized independently of the hospital. In several rural locations, ambulance services cover small populations. Here, they consist of one or two ambulances that are usually privately administered. However, private providers have established larger service units in some counties.

Participants in this study included officers, middle managers and managers who reported ambulance work to be more than 50% of their workload. The term "operational ambulance personnel" is used to describe these respondents.

Ambulance personnel received no formalized education until 1997. Thereafter, ambulance education consisted of a four-year education course at high-school level (including a two-year apprenticeship). Those who have achieved this qualification can apply for two-year part-time paramedic education at college level. Hence, the educational background of the ambulance personnel in Norway ranges from those with no formal education through to workers with formal ambulance education at high school or college level. Some of the ambulance personnel are also nurses or auxiliary nurses. Educational level was not related to any of the outcome variables and was therefore not applied as an independent variable. In April 2005, questionnaires were distributed to the chiefs of ambulance services in all 19 ambulance regions in Norway. All chiefs agreed to distribute the questionnaire to all ambulance personnel in the ambulance stations within their regions. This procedure was chosen because no central national register covering all employed ambulance personnel in Norway was available. Respondents were given an identification number in order to enable a follow-up one year later. Two follow-up reminders were distributed through the ambulance chiefs, and the two major worker union organizations encouraged their members to answer the questionnaire in their homepages and their membership journals. Respondents gave their consent to participate in the study by returning the questionnaire. The Regional Committee for Medical Research Ethics, Southern Norway, reviewed and advised the project.

In total, 3200 questionnaires were distributed. Based on reports from four of the ambulance chiefs, 64 ambulance personnel were excluded because they were no longer in service. In total, 1286 persons returned questionnaires (41%). Unfortunately, we were not able to get fully updated address lists from the other ambulance chiefs. Thus, the real response rate was probably higher than 41%. Responses were obtained from all ambulance regions. The response rates in the different regions ranged from 31% to 59%, median 41.5%, with the exception of one region with the low response rate of 10%. Overall, 53.4% of the respondents reported to work in rural settings (defined as serving a population with fewer than 20,000 inhabitants), and 46.6% reported to work in an urban setting (defined as serving a population with more than 20,000 inhabitants. Overall, the data show that we have rather good representation of ambulance personnel on a national.

Analysis of variance (ANOVA) was used to compare mean levels of the included health variables, and logistic regression was used to compare the level of help-seeking, and to test the assumption that the bivariate associations were similar in those who responded in the main round and those who responded after one or two reminders. There were no significant differences in descriptive scores, and no significant interactions between the bivariate associations and time of response.

Of the respondents, 1180 were operational ambulance workers (> 50% of their working time). Of these, 76.8% were men and 23.2% women. Age ranged from 18 to 66 years and the mean age was 36.8 (SD 9.3) years. The mean age for men was 37.6 (9.0) years, which was significantly higher than the mean age for women of 33.8 (9.6) (*p <*0.001).

### Reference sample

Each member of the population of Nord-Trøndelag County of Norway, aged 20 or older, was sent an initial questionnaire and an invitation to participate in HUNT 2 in 1995–97. In total, 92,100 people aged between 20 and 89 years were invited to participate in this study (initial questionnaire, including the HADS items). Of these, 65,648 (71%) attended a physical examination, where they received a second set of questionnaires (including the help-seeking items), of which 52,814 were completed. The general characteristics of the participants were fairly representative of the Norwegian population. Details on the data collection procedure and the characteristics of the study population have been published elsewhere [15]. In the present study, only those who were full-time employees and between 20 and 60 years old were included (N = 31,987). Of these 47% were men and 53% were women. Age ranged from to 20 to 60, with a mean age of 41.1 (SD = 10.3). The mean age was not significantly different for men and women. The questions on anxiety and depression were asked in the initial questionnaire (N = 31,618), and the questions regarding help-seeking were asked in the second set of questionnaires (N = 25,919).

### Instruments

Help-seeking was measured by one question: "Have you, during the last 12 months, contacted any of the following health professionals?" The response was "yes" or "no" on each of 10 alternatives: general practitioner, occupational health practitioner, physiotherapist, chiropractor, specialist in private practice, hospital physician with or without admission, other physician, psychologist/psychiatrist and homoeopathist. Four items (specialist in private practice, hospital physician with or without admission and other physician) were combined into the category "other physician." "Physiotherapist" and "chiropractor" were treated separately in the comparative analyses and combined in the ambulance specific analyses. The item "psychologist/psychiatrist" was only available in the ambulance sample. The item "homoeopathist" was omitted from the analyses because of the low number of respondents. The responses were not mutually exclusive.

Anxiety and depression symptoms were measured using The Hospital Anxiety and Depression scale (HADS) [16] and divided into a depression subscale (seven items) and an anxiety subscale (seven items). Each item is scored from 0 to 3. Forms with at least five of the seven items filled in on either subscale were included, and the score was computed as the mean of valid responses multiplied by seven. Principal component analysis supported a two-factor solution, anxiety (α = 0.8) and depression (α = 0.7), in both samples. Optimal balance between sensitivity and specificity for HADS as a screening instrument has been most frequently reported at a cut-off score of 8 or more [17], and this was chosen in the present study.

Data on sleep disturbance were obtained using four items from the Karolinska Sleep Questionnaire [18]. These items has been used as indicators of "disturbed sleep" in other studies [19,20]. Principal component analysis supported a one-factor solution: disturbed sleep ("difficulties falling asleep", "repeated awakenings", "premature awakening" and "disturbed/restless sleep") (α = 0.8). The items are scored on a five-point rating scale ranging from never (0) to always/everyday (4). The score was computed as the mean of valid responses, and the criterion for "disturbed sleep" symptoms was set at 3.5 [18].

The subjective experience of health was assessed by a 10-item version of the Subjective Health Complaint (SHC) questionnaire [21]. The items are scored on a four-point rating scale ranging from no complaints (0) to serious complaints (3). Each complaint is also scored for duration (number of days) during the last 30 days, but this information was not considered in the present analysis. "Somatic complaints cases" was defined as a score ≥ 2 on at least one of the 10 items yielding a prevalence of 43.6% [22]. This cut-off was chosen in order to allow comparisons with other samples (e.g., Norwegian police and physicians). Cases at single item level was (men/women): Shoulder (13.7%/18.2%), upper back (9.4%/10.9%), low back (22.1%/22.1%), neck (14.7%/20.7%), arm (7.0%/8.3%), leg pain during physical activity (4.6%/4.5%) and headache/migraine (10.5%/14.2%), chest (0.8%/0.8%), digestive problems (6.8%/7.1%) and vertigo (1.7%/1.1%). Consistent with previous studies [23], seven of the items (shoulder, upper back, low back, neck, arm, leg pain during physical activity and headache/migraine) resolved to a single factor defined as "musculoskeletal pain" (α = 0.7). The score was computed as the mean of valid responses, and used as a continuous variable in the regression analyses.

The Need for Recovery after Work Scale comprises 11 dichotomous items [24]. Typical items of this scale are: "At the end of a working day I am really feeling worn-out" and "I find it hard to relax at the end of a working day". The score was calculated by adding the individual's scores on the 11 items. Higher scores indicate a higher degree of need for recovery after work. This measure has been found reliable and valid, and it contains items that ask for the effect of the working day [25].

Gender was coded with men as a reference category. Age was used as a continuous variable with a one-unit change representing 10 years.

### Statistics

Means and frequencies were used to describe the data in the present study, and *t *tests and χ^2 ^tests were used to test for differences across samples. Principal component analysis with varimax rotation was used to check the factor structure of the instruments. Unianova analyses were used to compare mean scores across samples, adjusted for age. Logistic regression analyses were used to estimate the prevalence of depression and anxiety symptoms, adjusted for age and gender (separate analyses), and their relationship to help-seeking in the two samples. Further, simultaneous effects in the ambulance sample were estimated by logistic regression. The assumption of linearity in the logit held approximately for all independent health variables. These variables, together with age, were treated as continuous variables and standardized in the present analyses.

## Results

As can be seen from Table 1, the mean level of anxiety symptoms in the ambulance sample was significantly lower for both men (3.5 vs. 3.9, *P <*0.001) and women (4.0 vs. 4.4, *P <*0.05) and the mean level of depression symptoms was significantly lower for men (2.3 vs. 2.8, *P <*0.05) but not for women (2.9 vs. 3.1, *P = *0.22), when compared with the reference population adjusted for age. However, there was no significant difference between the ambulance sample and the general population in the prevalence of anxiety and depression cases (cut-off > 8).

**Table 1:** Description of the dependent and independent variables, and age adjusted estimates on anxiety and depression symptoms in the two samples

		Ambulance sample		General working population sample	
**A. Categorical variables**		**%**	**N**	**%**	**N**
Gender	Women	23.2	273	53.1	15017***
	Men	76.8	902	46.9	16969
	Total		1175		31986
		**% (95%CI)**		**% (95%CI)**	
Anxiety symptoms Cut ≥ 8	Men	9.8	89	11.8	1760 ns
	Women	13.6	37	16.4	2745 ns
*(age adjusted estimates)*	*Men*	*9.8 (7.8–11.8)*		*11.8 (11.3–12.3)*	*ns*
	*Women*	*14.4 (9.9–18.6)*		*16.4 (15.8–16.9)*	*ns*
Depression symptoms Cut ≥ 8	Men	7.3	65	7.5	1123 ns
	Women	3.7	10	7.1	1188 *
*(age adjusted estimates)*	*Men*	*8.0 (6.2–9.8)*		*7.0 (6.6–7.4)*	*ns*
	*Women*	*4.6 (2.1–7.2)*		*6.7 (6.3–7.1)*	*ns*
					
**B. Continuous variables**		**Mean**	**SD**	**Mean**	**SD **
AGE		36.8	9.3	41.1	10.3 ***
Anxiety symptoms (0–21)	Men	3.5	2.9	3.9	3.0 ***
	Women	4.0	3.0	4.4	3.3 *
*(age adjusted estimates)*		*3.5 (3.3–3.7)*		*3.9 (3.9–4.0)*	***
		*4.0 (3.6–4.4)*		*4.4 (4.4–4.5)*	*
Depression symptoms (0–21)	Men	2.7	2.8	3.1	2.8 ***
	Women	2.3	2.4	2.9	2.7 **
*(age adjusted estimates)*	*Men*	*2.9 (2.7–3.1)*		*3.1 (3.1–3.2)*	**
	*Women*	*2.7 (2.3–3.0)*		*2.9 (2.8–2.9)*	*ns*
					
**C. Ambulance sample (only)**					
Musculoskeletal pain (0–3)	Men	3.3	3.0		
	Women	3.9**	3.2		
Disturbed sleep	Men	0.4	0.8		
	Women	0.4	0.9		
Need for Recovery	Men	2.5	2.9		
	Women	2.4	2.7		

*P < 0.05; **P < 0.01; ***P < 0.001; Note. Level of significance was tested across samples and within gender, adjusted for age Adjusted estimates on anxiety and depression symptoms are presented in cursive.

As shown in Table 2, general practitioners were most often contacted by both groups but were contacted significantly less often by ambulance personnel than by the reference population, both for men (62% vs. 54%, *P <*0.001) and women (80% vs. 65%, *P <*0.001). Similar results were found for occupational health practitioners, who were contacted by 3% of ambulance personnel but by 24% of the reference population. However, ambulance personnel more often visited the chiropractor than did the reference population (11.7% vs. 4.5%). In total, 2.3% (3.5% of women and 1.9% of men, *P *= 0.13) had contacted a psychologist/psychiatrist. In the adjusted model, being an ambulance employee was negatively associated with visiting a general practitioner (OR 0.7, 95% CI 0.6–0.8), a physiotherapist (OR 0.7, 95% CI 0.6–0.9) and an occupational health practitioner (OR 0.1, 95% CI 0.05–0.11), and was positively related to visiting a chiropractor (OR 2.5, 95% CI 2.0–3.2). Anxiety and depression symptoms were positively related to more help-seeking from all professions, except that depression symptoms were not significantly associated with seeking help from chiropractors or occupational health practitioners. Two-way interactions between the sample and depression and anxiety symptoms, and three-way interactions with gender as the third variable, were tested but were not significant. Being female was associated with more help-seeking from all professions. Higher age was positively related to help-seeking from all professions except general practitioners.

**Table 2:** Help-seeking during last 12 months among ambulance personnel compared to a general working population

	General practitioner		Other physician		Physiotherapist		Chiropractor		Occupational health practitioner	
**Crude prevalences**	Gen	Amb	Gen	Amb	Gen	Amb	Gen	Amb	Gen	Amb
All	71.9	56.5 ***	32.7	27.2 ***	16.4	11.7 ***	4.5	11.7 ***	24.4	2.8 ***
Men	62.1	53.7 ***	28.7	25.0 *	13.5	9.5 **	5.2	10.9 ***	32.2	2.8 ***
Women	79.9	65.4 ***	36.1	34.1 ns	18.8	18.8 na	4.0	14.1 ***	17.5	2.9 ***
										
**Adjusted model**	OR (95% CI)		OR (95%CI)		OR (95% CI)		OR (95% CI)		OR (95% CI)	
Constant	1.5		0.4		0.1		0.1		0.5	
Ambulance sample	0.7 (0.6–0.8) ***		0.9 (0.8–1.1) ns		0.7 (0.6–0.9) *		2.5 (2.0–3.2) ***		0.1 (0.05–0.11)***	
Women	2.4 (2.3–2.5) ***		1.4 (1.3–1.5) ***		1.5 (1.4–1.6) ***		0.8 (0.7–0.9) ***		0.4 (0.4–0.5) ***	
Age	1.0 (0.9–1.0) ns		1.1 (1.1–1.2) ***		1.3 (1.2–1.3) ***		1.3 (1.3–1.4) ***		1.3 (1.3–1.4) ***	
Anxiety symptoms^+^	1.7 (1.6–1.9) ***		1.4 (1.3–1.6) ***		1.4 (1.3–1.6) ***		1.2 (1.0–1.5) ***		1.1 (1.1–1.3) ***	
Depression symptoms^+^	1.4 (1.3–1.6) ***		1.4 (1.2–1.5) ***		1.3 (1.2–1.5) ***		1.2 (0.9–1.5) ns		1.0 (0.9–1.2) ns	

*P < 0.05; **P < 0.01; ***P < 0.001; ^+ ^These scores were transformed into z-scores. Age refers to 10 year age bands Abbreviations. Gen = the general working population sample and Amb = the ambulance sample; Note. Two-way interactions with occupation were tested for anxiety and depression symptoms, and also three-way interactions with gender as a third variable were tested. None of these interactions were significant (not shown).

As can be seen from Table 3, the level of somatic complaints, disturbed sleep and need for recovery were significantly higher among ambulance personnel defined as anxiety or depression cases, compared with those without these levels of symptoms. The level of "musculoskeletal pain" was 43.6% in the total sample compared with 69.4% among anxiety cases and 75.7% among depression cases, and the prevalence of disturbed sleep was 6.3%, compared with 21.1% for anxiety and 31.1% for depression cases. The mean level of "need for recovery after work" was 2.5 in the total sample compared with 5.4 among anxiety cases and 6.2 among depression cases.

**Table 3:** Symptoms of disturbed sleep, musculoskeletal pain and need for recovery among operational ambulance personnel with anxiety and depression symptoms

		Musculoskeletal pain	Disturbed sleep	Need for recovery
		Prevalence (95%CI)	Prevalence (95%CI)	Mean (95%CI)
Total sample	all (n = 1161)	43.6 (41.1–46.9)	6.3 (4.9–7.7)	2.5 (2.3–2.7)
	men (n = 889)	44.9 (41.7–48.3)	5.9 (4.3–7.4)	2.5 (2.3–2.7)
	women (n = 272)	43.2 (37.1–48.8)	7.8 (4.6–11.0)	2.4 (2.1–2.8)
				
HADS Anxiety ≥ 8	all (n = 126)	69.4 (60.9–77.1) χ^2^***	21.4 (14.3–28.8) χ^2^***	5.4 (4.9–5.8) t-test
	men (n = 89)	69.7 (60.5–79.5) ***	23.6 (14.8–32.4) ***	5.9 (5.3–6.4) ***
	women (n = 37)	68.6 (54.1–83.9) **	16.2 (4.3–28.1) *	4.3 (3.4–5.2) ***
				
HADS Depression ≥ 8	all (n = 85)	75.7 (66.9–85.1) ***	31.1 (20.5–41.6) ***	6.2 (5.6–6.8) ***
	men (n = 65)	76.6 (66.8–87.2) ***	28.1 (17.1–39.2) ***	6.3 (5.6–7.0) ***
	women (n = 10)	70.0 (41.6–98.4) ns	50 (19.0–80.1) ***	5.4 (3.6–7.1) **

*P < 0.05; **P < 0.01; ***P < 0.001. Chi-square tests were performed for those with anxiety or depression symptoms against those without anxiety and depression symptoms, respectively.

As shown in Table 4, all types of health problems were significantly bivariately associated with more help-seeking, except for depression and anxiety symptoms, which were not significantly related to visiting a physiotherapist or a chiropractor. Bivariate associations between independent variables were ranging between Pearson's r = .36 and r = .56. Thus, multicollinearity was not deemed problematic given the large sample size. In the adjusted model, musculoskeletal pain was significantly related to more help-seeking from all health professions, except for a psychologist/psychiatrist. Symptoms of disturbed sleep were significantly related to more help-seeking from a general practitioner, and disturbed sleep was the only health indicator that was independently associated with visiting a psychologist/psychiatrist (OR 1.6, 95% CI 1.0–2.7). Need for recovery was significantly related to visiting a physiotherapist or a chiropractor (OR 1.2, 95% CI 1.9–1.5). Anxiety and depression symptoms were not, however, significantly related to more help-seeking in the adjusted model. Men reported significantly lower levels of help-seeking from all professions, except for psychologists/psychiatrists, and age was not related to help-seeking in the adjusted model.

**Table 4:** Help-seeking during last 12 months among Norwegian ambulance personnel

	General practitioner	Other physician	Physiotherapist/Chiropractor	Psychologist/Psychiatrist	Occupational health practitioner
**Bivariate model**	**OR (95%CI)**	**OR (95%CI)**	**OR (95%CI)**	**OR (95%CI)**	**OR (95%CI) **
Anxiety symptoms^+^	1.4 (1.3–1.6) ***	1.3 (1.2–1.5) ***	1.1 (0.9–1.3) ns	1.8 (1.3–2.4) ***	1 (0.7–1.5)
Depression symptoms^+^	1.3 (1.1–1.4) ***	1.2 (1.1–1.4) **	1.1 (0.9–1.2) ns	1.7 (1.3–2.3) ***	0.8 (0.5–1.2)
Musculoskeletal pain^+^	1.7 (1.5–1.9) ***	1.5 (1.4 1.8) ***	1.9 (1.6–2.2) ***	1.7 (1.3–2.4) ***	0.7 (0.4–1.1)
Disturbed sleep^+^	1.6 (1.4–1.8) ***	1.3 (1.2–1.5) ***	1.2 (1.0–1.4) **	2.2 (1.5–3.3) ***	1 (0.7–1.4)
Need for recovery^+^	1.4 (1.3–1.6) ***	1.4 (1.2–1.5) ***	1.3 (1.1–1.5) ***	1.6 (1.2–2.3) ***	0.7 (0.4–1.0)
Women	0.6 (0.5–0.8) **	0.6 (0.5–0.9) **	0.6 (0.4–0.8) **	0.5 (0.2–1.2) ns	1 (0.4–2.5)
Age	1(0.9–1.1)	1.1 (1.0–1.3) ns	1.2 (1.0–1.4) ***	1.2 (0.8–1.8) ns	1.6 (1.1–2.3) *
**Adjusted model**	**OR (95%CI)**	**OR (95%CI)**	**OR (95%CI)**	**OR (95%CI)**	**OR (95%CI) **
Constant	1.8	0.4	0.2	0.02	0.01
Anxiety symptoms^+^	1.1 (0.9–1.3)	1.1 (0.9–1.3)	1 (0.8–1.2)	1.1 (0.7–1.8)	1.6 (1.0–2.5) *
Depression symptoms^+^	0.9 (0.8–1.1)	1 (0.8–1.1)	0.8 (0.7–1.0) *	1.2 (0.8–2.0)	0.7 (0.4–1.3)
Musculoskeletal pain^+^	1.5 (1.3–1.7) ***	1.4 (1.2–1.6) ***	1.9 (1.6–2.3) ***	1.3 (0.9–1.9)	0.7 (0.4–1.1)
Disturbed sleep^+^	1.2 (1.0–1.4) *	1.1 (0.9–1.3)	0.9 (0.7–1.1)	1.9 (1.1–3.2) *	1.2 (0.8–1.8)
Need for recovery^+^	1.1 (0.9–1.3)	1.1 (1.0–1.3)	1.2 (1.9–1.5) *	1 (0.6–1.5)	0.7 (0.4–1.1)
Women	0.7 (0.5–1.0) *	0.7 (0.5–0.9) *	0.6 (0.4–1.0) *	0.6 (0.2–1.6)	1 (0.4–2.6)
Age	1 (0.8–1.1)	1.1 (0.9–1.3)	1.2 (1.0–1.4)	1 (0.6–1.7)	1.8 (1.2–2.7) **

-2 log likelihood	1377.9	1140.7	913.2	195.4	254.7

*P < 0.05; **P < 0.01; ***P < 0.001; ^+ ^These scores were transformed into z-scores. Age refers to 10 year age bands. Note. Two-way interactions with anxiety, depression and the other health indicators were tested. None of these interactions were significant (not shown).

## Discussion

The mean level of anxiety and depression symptoms was lower among ambulance personnel than in the general working population. This is an unexpected finding because the comparison group consists of the general working population and the healthy worker effect is therefore probably diminished. Being female in a male-dominated working environment such as the ambulance services does not seem to be a risk factor for mental health problems among ambulance women. In general, there were small and non-significant differences between men and women in the ambulance sample, and the relative gender differences were similar to those reported in the general working population. The prevalence rates of anxiety and depression cases (11% and 7.2%, respectively) among operational ambulance personnel were similar to those reported for Norwegian police (11.8% and 8.2%, respectively) [8] but were substantially lower than the high prevalence rates (> 20%) that have been reported in some studies of ambulance personnel [1,2,26,27]. However, none of the previous ambulance studies was nationwide, and other studies have reported lower prevalence rates (2.1%) [28] and a lower mean level of anxiety and depression in ambulance workers compared with a normative sample of working males in the USA [29].

The present data support the hypothesis that ambulance personnel in general are less likely to seek help than the general working population. These differences were significant when adjusted for age, gender and level of depression and anxiety symptoms. It is noteworthy that as little as 3% of ambulance workers had visited an occupational health practitioner, compared with 24% of the general working population. Higher age was significantly related to visiting an occupational health practitioner, which might reflect a higher level of mistrust and concern about anonymity among younger workers or a lack of information about these services to new employees. Overall, these findings partly support the assumption that health care workers may be more reluctant to seek professional help, but they do not support the hypothesis that they are especially reluctant to seek professional help for mental health problems.

The level of somatic complaints cases was relatively high (43.6%), which is similar to that reported for a nationwide sample of Norwegian police (40.7%) [8] and considerably higher than that reported for Norwegian physicians (29.5%) [22]. However, the chosen cut-off value for "cases" is low and has not been validated, hence the results should be interpreted with caution. Prevalence rates at item level were available from the general population in Norway [30]. Ambulance men report a higher prevalence of pain in neck (14.7% vs. 11.2%), upper back (9.4% vs. 6.0%), and lower back (22.1% vs. 14.0%) compared to that of the males in the general population. Ambulance women, on the other hand, report a lower prevalence on all musculoskeletal pain items compared to that of women in the general population, but relative high level of pain in neck (20.7% vs. 14.7) and shoulder (18.2% vs. 13.7) compared to that of ambulance men. The general population sample included people who were not employed and in a wider age range (aged 15–84 year) than in the present, and the comparison should be interpreted with caution. However, given that that a general population sample most likely is less healthy than a sample drawn from an expectedly health working population, the level of musculoskeletal pain symptoms, particularly among male ambulance personnel, may be considered relatively high. The prevalence of disturbed sleep symptoms (6.3%) was similar to that reported in a study encompassing 40 companies in central Sweden [18]. Unfortunately, no Norwegian reference data were available. These prevalence rates are low compared with the 30% reported in a study of ambulance personnel in Sweden. However, the Swedish study only included one item on sleep, and cases were defined as respondents reporting "sleeping problems sometimes or often [31]."

Musculoskeletal pain was most consistently related to more help-seeking from all health professions, except for occupational health practitioners. Furthermore, ambulance personnel visited a chiropractor more often than did the reference population (11.7 vs. 4.5). Although it is generally known that depressive symptoms might be hidden and shown as somatic complaints [32], neither depression nor anxiety were bivariately associated with ambulance workers visiting a chiropractor/physiotherapist. In contrast, 2.3% had visited a psychologist/psychiatrist. In the adjusted model, only symptoms of disturbed sleep were significantly associated with visiting a psychologist/psychiatrist. Given the relatively low level of anxiety and depression symptoms, this might indicate adequate help-seeking, although reluctance towards seeking help from a psychologist/psychiatrist cannot be ruled out.

In the exploration of the relationship between different types of health problems, neither symptoms of anxiety nor depression was significantly associated with more help-seeking in the adjusted model. This could be seen as a consequence of the higher proportion of symptoms of musculoskeletal pain, disturbed sleep and need for recovery among those with anxiety and depression symptoms, which is consistent with what has been reported in the general literature [33,34]. The prevalence of somatic complaints was more than 1.6 times greater (69.4/43.6%), the prevalence of disturbed sleep more than 3.4 times greater (21.4/6.3%) and the mean level of need for recovery 2.2 times greater (5.4/2.5) among anxiety and depression cases compared with the total sample. The distinction between these conditions can be a challenge not only in epidemiological surveys but also in clinical practice. We did not find, however, any support for the assumption that the association between anxiety or depression symptoms and help-seeking might depend on the co-occurrence of other health symptoms. Furthermore, males reported significantly lower levels of help-seeking from general practitioners, hospital physicians and physiotherapists, which is consistent with reports from studies of the general population [35] and Norwegian police [8]. Higher age was bivariately related to help-seeking from a physiotherapist/chiropractor. In the adjusted model, this relationship was no longer significant and was mainly explained by the higher level of musculoskeletal pain among the older workers, which is in accordance with what has been found in other studies [12]. Otherwise, help-seeking was largely unaffected by age.

### Strengths and limitations

The major strength of this study is that it is the first large-scale nationwide study of operative ambulance. Furthermore, data on depression and anxiety symptoms, as well as help-seeking, were compared with a general working population in Norway. Some limitations of this study should be considered. The cross-sectional design prevents us from providing direct evidence of the direction of the reported relationships. Report bias may be a problem as, for example, anxiety and depressive symptoms are socially undesirable topics, particularly in a masculine milieu. Further, the response rate was relatively low, which may compromise the representativeness of the data, and raises the possibility that differences between the two groups are due to selection biases, particularly if the non-responders in ambulance workers had more anxiety and depression symptoms. However, there were no significant differences in descriptive scores, and no significant interactions between the bivariate associations and time of response. As late responders may resemble the non-responders [36,37], the lack of representativeness may not be a severe problem. Further, because of the problems in the questionnaire distribution, it is likely that the real response rate is substantially higher than the estimated proportion.

## Conclusion

Contrary to common beliefs, ambulance personnel reported significantly less anxiety and depression symptoms than did the general working population in Norway. Help-seeking was less frequent among ambulance personnel and this effect was independent of the level of anxiety and depression symptoms. The level of musculoskeletal pain and, accordingly, the level of help-seeking from a chiropractor were relatively high. More research should address the physical strains among ambulance personnel. Operational ambulance personnel might be reluctant to contact a psychologist/psychiatrist or an occupational health practitioner. The ambulance services may benefit from providing their employees with better information about their occupational health practitioner systems.

## Competing interests

The author(s) declare that they have no competing interests.

## Authors' contributions

TS, EH, ØE and BL jointly conceived the idea for the paper. TS performed the statistical analyses. All authors interpreted the data. TS drafted the manuscript. TS will act as guarantor for the paper. All authors approved the final manuscript.

## Pre-publication history

The pre-publication history for this paper can be accessed here:


